# Urolithins: The Colon Microbiota Metabolites as Endocrine Modulators: Prospects and Perspectives

**DOI:** 10.3389/fnut.2021.800990

**Published:** 2022-02-02

**Authors:** Ravindran Vini, Juberiya M. Azeez, Viji Remadevi, T. R. Susmi, R. S. Ayswarya, Anjana Sasikumar Sujatha, Parvathy Muraleedharan, Lakshmi Mohan Lathika, Sreeja Sreeharshan

**Affiliations:** ^1^Cancer Biology Division, Rajiv Gandhi Centre for Biotechnology, Thiruvananthapuram, India; ^2^Botany Department, Mount Carmel College, Bengaluru, India

**Keywords:** urolithin, estrogen receptor, selective estrogen receptor modulators, pomegranate (*Punica granatum* L.), PhytoSERM

## Abstract

Selective estrogen receptor modulators (SERMs) have been used in hormone related disorders, and their role in clinical medicine is evolving. Tamoxifen and raloxifen are the most commonly used synthetic SERMs, and their long-term use are known to create side effects. Hence, efforts have been directed to identify molecules which could retain the beneficial effects of estrogen, at the same time produce minimal side effects. Urolithins, the products of colon microbiota from ellagitannin rich foodstuff, have immense health benefits and have been demonstrated to bind to estrogen receptors. This class of compounds holds promise as therapeutic and nutritional supplement in cardiovascular disorders, osteoporosis, muscle health, neurological disorders, and cancers of breast, endometrium, and prostate, or, in essence, most of the hormone/endocrine-dependent diseases. One of our findings from the past decade of research on SERMs and estrogen modulators, showed that pomegranate, one of the indirect but major sources of urolithins, can act as SERM. The prospect of urolithins to act as agonist, antagonist, or SERM will depend on its structure; the estrogen receptor conformational change, availability and abundance of co-activators/co-repressors in the target tissues, and also the presence of other estrogen receptor ligands. Given that, urolithins need to be carefully studied for its SERM activity considering the pleotropic action of estrogen receptors and its numerous roles in physiological systems. In this review, we unveil the possibility of urolithins as a potent SERM, which we are currently investigating, in the hormone dependent tissues.

## Introduction

Selective estrogen receptor modulators (SERMs) are non-steroidal compounds that bind to estrogen receptors and can act like estrogen or be a partial agonist or antagonist with mixed activity depending on the tissue it acts. Tamoxifen and raloxifen are the most commonly used SERMs in treating breast cancer, which is often observed to exert side effects in hormone dependent-tissues. Tamoxifen, for instance, though osteoprotective like estrogen (1, 2), is reported to increase the uterine weight, endometrial cancer, stroke, and pulmonary embolism (3). A drug that has protective effects in estrogen-dependent tissues and prevents its deleterious effects would serve as an ideal SERM (4). Overcoming the side effects of synthetic SERMs is highly coveted for treating ER-positive breast cancer and other hormone related disorders. Hence, there has been an extensive search for alternatives from plant-based molecules with structural and functional resemblance to estrogens such as phytoestrogens. These are present in soy, grains, vegetables, and berries and are often metabolized by microbiota to form compounds, with or without having an estrogen-like activity (5). Many times, these phytoestrogens are metabolized by gut microbiota, which often have a stronger activity attributed to their higher lipophilicity, leading to a better absorption, and a higher affinity with estrogen receptors (6). These metabolites can, in turn, modulate the gut microbiota rendering a bidirectional relationship (7, 8). For instance, S-equol derived from daidzein by an intestinal bacterial metabolism also displays a profile like that of the daidzein's, and is of clinical importance (6, 9). Notably, many of the phytoestrogens like genistein, coumestrol, and liquiritigenin display more affinity toward estrogen receptor β (ERβ) than to estrogen receptor α (ERα), but the implications and underpinnings of these differences remain elusive (10). It is probable that those tissues where ERβ is critical, such as ovary, prostate, lung, cardiovascular, and central nervous systems (CNSs) (11), might be more influenced by these compounds.

Pomegranate has been known to have extensive medicinal properties which have been attributed to its constituents, working individually or in combination (12, 13). We have also demonstrated that pomegranate can act as SERM (4). Pomegranate is rich in ellagitannins and ellagic acid, which is further metabolized to urolithins by a specific colon microbiota. Ellagitannins and ellagic acid are also found in certain berries and nuts like walnuts and pecans. However, there is lot of inter-individual variability in the presence and abundance of ellagic acid and urolithins in plasma, urine, and feces of the individuals consuming ellagitannin-rich food, which could be primarily due to the presence, absence, or abundance of some specific microbiota (14). Urolithin family, characterized by a chemical structure containing α-benzo-coumarin scaffold, majorly include Urolithin A (UA),Urolithin B (UB), Urolithin C (UC), Iso-Urolithin A (Iso-UA), the recently discovered Urolithin M7R, Urolithin CR, and Urolithin AR (15). The main metabolites found in plasma, tissues, and excreted in urine and feces include UA, UB, and Iso-UA, which are subsequently absorbed and metabolized into their corresponding phase II conjugates (glucuronides or sulfates) and can persist in the bloodstream up to 3–4 days after the intake (13). Urolithins are understood to be actively glucuronidated in the large intestinal enterocytes before entering the bloodstream. Their maximal concentrations in the plasma can reach up to 35 μM for glucuronide and 8-O-glucuronide, 0.745 μM for Iso-UA 3-O-glucuronide, and 7.3 μM for and urolithin B 3-O-glucuronide (16). Repeated consumption of ellagitannin-containing food products can significantly increase the concentration of these conjugates in urine (16). Among the urolithins, UA and its conjugates are found at the highest concentrations in human plasma ranging from 0.024 to 35 μM. Urolithins are detected in high concentrations in the colon and can also reach the systemic tissues such as the prostate and mammary gland (13). Direct supplementation with UA significantly increases plasma levels and provides more than six-fold exposure to UA vs. pomegranate juice (17).

Accumulating evidence suggests urolithins have extensive health benefits (18). It has been shown that urolithins have estrogenic and antiestrogenic activity (19) *via* competitive binding assays, proliferation assays (20), and transactivation assays (21), and is predicted to bind to ERα in a similar orientation as that of the estrogen (21). However, the conjugates of urolithins, which reach the breast tissue after the ellagitannin intake, apparently lack estrogenic and anti-estrogenic activity (22). The ellagic acid from which urolithins are derived have also been reported to exhibit estrogenic activity at low concentrations (10^−7^ to 10^−9^ M), *via* ERα, whereas it was a complete estrogen antagonist *via* ERβ (23), though another investigation demonstrates absence of any estrogenicity or antiestrogenic activity in ellagic acid (21). Notably, UA and UB have been known to inhibit aromatase (24) and 17β-hydroxysteroid dehydrogenases (25), which are the enzymes critical for estradiol synthesis. All these pointedly illustrate the ability of urolithins to have estrogenic/anti-estrogenic or SERM-like profile. Briefly, estrogenic chemicals are those that can directly activate or inhibit estrogen action or can indirectly modulate its action. These can act as endocrine disruptors, which are defined as “an exogenous agent that interferes with the production, release, transport, metabolism, binding, action, or elimination of natural hormones in the body responsible for the maintenance of homeostasis and the regulation of developmental processes” (26). The SERMs are cornerstone strategy to treat breast cancers, infertility problems, postmenopausal problems like hot flashes, osteoporosis, and for hormone replacement therapy. Hence, this implies that it is highly beneficial if urolithins can be employed as SERMS or as estrogenic compounds, with minimum side effects. Of note, urolithins are considered safe according to toxicity studies (27, 28), and, most importantly, UA has been recently recognized by food drug administration (FDA, USA) as GRAS (Generally Recognized As Safe) for its use as an ingredient. In this review, we explore the prospect of urolithins to act as endocrine modulators/disruptors by consolidating and connecting the existing body of evidence that underpins the health benefits of urolithins in hormone-dependent tissues and propose that it can act as an estrogen agonist, or antagonist or as SERMs, and detail its potential to modulate the hormone related pathways. The following sections unfold the benefits of urolithins in tissues where estrogen has a remarkable role. This includes its anti-inflammatory potential, cardiovascular benefits, breast, endometrial and prostate cancer protection, bone, muscle, and cognitive health where estrogen receptors are abundant and play a pivotal role.

## Colon Bacteria: The Cooks Who Brought Out the Delicacy

Right from the mode of delivery to the kind of feeding pattern, the environment and the dietary pattern mold the gut microbiota. The response to diet depends on the type of bacteria that inhabits the gut and also interaction of the host microbe. This process is cyclic and inter-dependent (29). Similarly, the nutritional availability and, hence, therapeutic, or preventive effect of a diet can vary with microbiome features and their abundance. The inter-individual variability in the presence and abundance of ellagitannins/urolithins in plasma and urine samples after consumption of ellagitannin rich food was suggestive of their microbial origin in colon. Speculations were put to rest when results from Cerda et al. (30) confirmed that urolithins and its types correlated with the type of the fecal bacteria. This affirmed the microbial origin of urolithins in humans and explains the difference in the therapeutic and nutritional effects of pomegranate and berries that have similar rich composition of ellagitannins. This discovery led to the active research in identification of the microorganisms which can convert ellagitannins/ellagic acid to different types of urolithins. Selma et al. identified intestinal bacterial species from human feces, namely, *Gordonibacter urolithinfaciens* and *Gordonibacter pamelaeae* (31), belonging to the family Eggerthellaceae, which transformed the ellagic acid into UC, and another strain CEBAS 4A4, belonging to a new genus from the same family, could produce Iso-UA (32) under anaerobic condition leading to the categorizing of individuals into three metabotypes, according to the gut microbiota composition (33). After ingestion of ellagitannin-rich food products, individuals without urolithins production belonged to metabotype 0, with UA production as the unique final product that fits into metabotype A, and UB and/or Iso-UA belongs to metabotype B (14, 18). Thus, this difference in microbiota composition would further influence the health benefits associated with ellagitannin-rich food (34). Notably, urolithins have been found in breast milk of mothers, who consume ellagitannin rich walnut, and it resembles Urolilithin metabotypes of the mothers as well (35). Investigations on how these bacteria can improve the metabolism of an ellagitannin-rich food, how would it further bring about health benefits, and an examination of its safety aspects are vital before considering them as a potential probiotic.

## Anti-Inflammatory Activity and Cardiovascular Protection

Ellagitannins and urolithins have antioxidant (36), anti-inflammatory (37–41), and immunomodulatory properties (42). Urolithins inhibit NF-kb in colon fibroblast (43), in osteoarthiritic models (44), and in rat primary chondrocytes (45), but whether they act *via* estrogen receptors or membrane receptors like estrogen do, is not studied estrogens (46) is not studied. The UA and its metabolites have been shown to be protective in cardiac health (47, 48). *In vivo* studies with streptozotocin-induced type-1 diabetes rats demonstrated that urolithins administration reduced the myocardial expression of the pro-inflammatory cytokine fractalkine, thus, improving the cardiac performance (49). Furthermore, urolithin B-glucuronide (UB-glu) could counteract trimethylamine-N-oxide-induced cardiomyocyte damage (48). Direct consumption of pomegranate has also shown the beneficial effects in the cardiovascular health (50–53). In our earlier study, we found a reduction in low density lipopolysaccharide (LDL) in the Swiss-albino mice after consumption of methanolic extract of pomegranate (PME), which was induced upon ovariectomy when compared to sham control (4).

Estrogens exert anti-inflammatory activity *via* the receptors like ERα, ERβ, and GPR-30 that are present on cardiac cells encompassing cardiomyocytes, fibroblasts, vascular endothelial, and smooth muscle cells (54), thus, being beneficial in cardiovascular diseases such as coronary heart disease, ventricular hypertrophy, atherosclerosis, etc., *via* nuclear or non-nuclear pathways (55, 56). The ERβ appears to have a substantial cardioprotective effect (54). However, whether the protective effects of the pomegranate components or of urolithins are caused by these receptors are not investigated. It would be worthwhile to unravel whether they show similar or varying profile and benefits in different categories such as in pre-, peri- and post-menopausal women, and whether pomegranate benefits correlate their metabotype and different hormonal phases in women.

## Breast Cancer

Our previous research showed that methanolic extract of a pomegranate peel reduced breast cancer proliferation by binding to estrogen receptor without affecting uterine weight, unlike estradiol or tamoxifen (4). A plethora of evidence points to preventive possibilities of pomegranate in breast cancer at its various stages and processes of cell survival (12). We had also reported that the PME can inhibit the proliferation induced by endogenous SERM 27 hydroxycholesterol (57). From the findings so far, urolithins are active molecules, but its availability and type are dependent on the gut microbiota and, hence, the extract may have limitations in its applicability. For the first time, Larossa et al. demonstrated in 2005 that UA and UB can act as “enterophytoestrogens,” exhibiting estrogenic activity in a dose-dependent manner without antiproliferative or toxic effects. Urolithins in combination with estrogen showed antiproliferative activity and anti-estrogenic activity and thwarted the proliferative activity of estrogen in the cell line models of human breast. The competitive binding assay showed that UA had much higher affinity to both ERα and ERβ than UB. The UA had a slightly higher affinity toward ERα than ERβ, with half maximal inhibitory concentration (IC50) values being at 0.40 and 0.75 μM. Skledar et al. (21) reported a comparatively higher half maximal effective concentration (EC50) value for ERα, i.e., 5.60 μM. However, the conjugated metabolites of urolithins lacked these activities (22, 58). Hence, it is speculated that the potential antiproliferative/cytotoxic, as well as estrogenic/antiestrogenic activities, in breast tissues would primarily depend on the metabolite formed in the specific tissues. It is also suggested that though conjugation may hinder the direct antiproliferative activity, there is a probability of a long-term tumor-senescent chemoprevention (22).

Urolithins have been documented to inhibit aromatase and proliferation of breast cancer cells stimulated by testosterone (24). Strikingly, UA and UB inhibit 17β-hydroxysteroid dehydrogenases (17β-HSD1) (25), an enzyme involved in dihydrotestosterone (DHT) inactivation and in the conversion of inactive estrone (E1) to estradiol (E2), and, hence, critical for estradiol synthesis. Of note, high 17β-HSD1 mRNA expression in patients with breast cancer correlates with a weak prognosis for breast cancer, thus, enhancing the breast cancer proliferation and invasion (59). Additionally, urolithins inhibit androgen receptor (60). These results point to its ability to act as anti-estrogenic in breast cancer. The UA also suppresses hyper-activated transglutaminase TGM2 which is one of the novel gene signatures expressed in metastatic cells that have undergone induction and reversion of epithelial–mesenchymal transition (EMT) and have induced metastasis (61). Also, urolithins can cross blood brain barrier and, hence, its potential in preventing breast to brain metastasis is worth investigating, since there is very less information on anti-estrogens or aromatase inhibitors that can cross blood brain barrier, which is a potential endocrine tissue (62, 63).

## Endometrial Protection

Endometrial cancer is the sixth most commonly occurring cancer in women (64). In premenopausal women, endometrial proliferation is driven by estrogen, whilst after menopause, peripheral tissues like adipose tissue takes over estrogen synthesis, which implies that obesity increases likelihood of endometrial cancer in the postmenopausal uterus (65). In this context, it is worthwhile to mention that the pomegranate and urolithins have been shown to have preventive roles in obesity (4, 66–68). Urolithins are found to inhibit human endometrial cancer cells in an *in vitro* study (20). It also modulated the expression of ERα-dependent genes like ERβ, PGR, pS2, and GREB1. The UA and UB exhibited antiproliferative activity in the human primary endometriotic cells and reduced the invasion and expression of Matrix metalloproteases (MMPs) and matrix adhesion receptor. Both UA and UB were found to decrease the viability and integrity of endometriosis spheroids (69). These studies are indicative of the ability of urolithin to have protective effects on the endometrium. Endometrial cancers are mostly hormone driven *via* estrogen receptors (70) and, hence, are often a side effect of SERMs like tamoxifen (71). Therefore, SERMs which do not activate endometrial proliferation is much desired in the present clinical context.If urolithins are further explored for their activities in hormone-dependent tissue, they can be exploited for their SERM activity. Hitherto, there are only *in vitro* evidence. It is important to consider that, within the body, the urolithins undergoes the phase-II conjugation (72) and reaches the systemic tissues. These conjugates have not been studied for antiproliferative activity in the endometrial cancer. Interestingly, the tissue level of the deconjugation of urolithin glucuronides has also been reported (72). Indeed, more concrete studies and evidence are needed to understand and to prove the real protective capacity of these compounds and their distribution with respect to endometrium and their combinatorial behavior with other synthetic SERMs.

## Prostate Wellness

In 2020, prostate cancer was the second most commonly occurring cancer in men, as well as the fifth leading cause of death among men (64). It is noteworthy to mention that prostate cancer has been reported to be driven by ERα, AR, and non-genomic estrogen signaling pathways mediated by orphan receptors like GPR30 and ERRα and, hence, phytoestrogens have been shown to have a beneficial role in prostate cancer (73, 74). Pomegranate and its metabolites, ellagic acid and urolithins, found concentrated in mouse prostate, colon, and intestinal tissues (75), prevent proliferation of prostate cancer (60, 76–79). Interestingly, it has been reported that the beneficial effects of pomegranate juice against prostate cancer may be exerted by urolithin glucuronides and dimethyl ellagic acid (38). Combinatorial treatment with urolithins and bicalutamide, a clinically used non-steroidal anti-androgen, used to treat prostate cancer (80), also showed antiproliferative effects on human prostate cancer cells. Antiproliferative effects of urolithins were more conspicuous in androgen-independent than in androgen dependent cells. Antiproliferative activity of urolithins was mediated by AR through AKT signaling pathway (81) and P53-MDM2 pathway (82). Although the possible role of estrogen receptor has been proposed in other studies (80), there is still no clear evidence. Apart from this, urolithins can strengthen muscles (83), which is an added advantage while treating prostate cancer by androgen deprivation, given that this therapy can cause muscle weakness (84). If this study proves or extends its applicability in humans, urolthins could be helpful in general wellness of prostate, as an SERM, an adjuvant, or for muscle strengthening. Pomegranate and its constituents, including its colonic metabolite, have shown propitious results in prostate cancer. These findings provide very interesting leads and points to the ability of the pomegranate metabolites for the prevention of prostate cancer recurrence (85). The chemopreventive potential of pomegranate ellagitannins, coupled with the finding that urolithin metabolites accumulate in prostate, suggests that pomegranate may be a prospective therapeutic formulation in prostate cancer. Major urolithin that accumulates in the prostate after ellagitannin consumption is UA and its metabolite Urolithin A glucuronide (UA-glu). However, the concentration of these are very low, that is, in the range of ng/g albeit urolithins reach micromolar level concentrations in the bloodstream (38). Hence, the *in vitro* evidence using supraphysiological concentrations of urolithins or unconjugated urolithins might not give a realistic data on whether these metabolites can be protective in prostate cancer. Studies should be undertaken to understand the physiological levels of urolithin, or its conjugates, that can accumulate in the prostate upon consumption of urolithins at its safe doses, and how is it beneficial in prostate cancer at these doses.

## Urolithins a Glimmer of Hope in Bone Health

Our earlier studies have shown that pomegranate has a protective role in osteoporosis (86) using MC3T3-E1 cells and ovariectomised Swiss–albino mice. The results indicated that the PME (80 μg/ml) has significantly increased the ALP (Alkaline Phosphatase) activity, in agreement with the findings of Spilmont (87), suggesting its role in modulating osteoblastic cell differentiation. This connotes its potential as a promising nutritional supplement in management of osteoporosis associated with menopause. The UA has been shown ameliorate intervertebral disc degeneration, a common cause of back pain (88) in a needle-puncture rat tail model via c-JUN and PI3K/Akt/NF-κB pathways. The UA inhibited the inflammatory molecules and debilitated the degradation of the extracellular matrix (ECM) induced by IL-1β. Both *in vitro* and *in vivo* evidence support its protective role in osteoarthritis (44). The question whether urolithins can act *via* estrogen receptors like estrogen (89) in these cells have not yet been explored. Evidence points to the potential of urolithins in promoting bone health, giving it an edge to be an ideal SERM.

## Neurodegenerative Disease

A plethora of studies using different models have adduced that estrogens play a vital part in protecting women against stroke and neurodegenerative diseases, though the mechanisms have not been fully elucidated. All the neural cells express estrogen receptors and the neuroprotective properties are, in part, attributed to the receptor activation in multiple cell types. Microglial cells, the major immune cells that inhabit the CNS, are regulated by estrogen, which, in turn, protects the neuronal functions and prevents neurodegeneration (90). This offers the prospect of selectively targeting estrogen receptors in the treatment of neurodegenerative conditions that comes with aging and menopause. Interestingly, PE is demonstrated to act against Alzheimer's and Parkinson's disease in many studies (91–95). Presence of urolithins in brain after consumption of pomegranate has also been reported (95). Urolithins were the only compounds, among 21 others, that is isolated from the extract that met the criteria required for the penetration of blood-brain barrier (BBB) permeability (94). The β-amyloid fibrillation was averted by urolithins in an *in vitro* study. In Alzheimer's model, UA imparts cognitive protection by protecting neurons from cell death, and by triggering neurogenesis *via* anti-inflammatory signaling. In addition, it inhibits monoamine oxidase (MAO) (96), an enzyme that inactivates monoamine neurotransmitters in neurological disorders, such as depression and Parkinson's disease (97). Although the neuroprotective effects of urolithins are reported, evidence is still weak, partly due to the lack of physiologically relevant studies using the circulating conjugated urolithins that might reach brain tissues, in a nutritional context. In 2017, González-Sarrías et al. (98) demonstrated that UA and Iso-UA, but not UB-glu, showed a slight attenuation of the H_2_O_2_-induced cytotoxicity in human-derived neuroblastoma SH-SY5Y cells. Another study showed that media from lipopolysaccharide (LPS)-BV-2 murine microglial cells co-culture cell model, treated with urolithins, preserve the SH-SY5Y cell viability (99), and protect neuroinflammation, although, methylated urolithins have not been detected in circulation in humans, so far. Thus, to date, only limited studies were performed with conjugated urolithins in the context of neuronal protection. Notably, SERMs like tamoxifen and raloxifene are known to regulate the functions of astrocytes, neurons, and microglia *via* ERα and the ERβ and G-protein coupled estrogen receptor (GPR30) (100). Hence, it would be interesting to unveil the potential of the urolithins to act as estrogen receptor modulators in the neuroimmune axes.

## Aging and Muscle Strength

Ryu et al. (83) found that feeding of *C. elegans* during entire lifetime, i.e., from eggs until death with 50 μM of UA, UB, UC, and Urolithin D (UD), has extended the lifespan by 45.4, 36.6, 36, and 19.0%, respectively, and was dose dependent. However, the treatment with ellagic acid had no effect. Mitophagy induced by urolithins in mammalian cells, *C. elegans*, and rodents culminated in the improvement of the overall health. The short duration of urolithin administration in young worms and mammalian cells reduced the mitochondrial content without affecting the maximal respiratory capacity. This proved that despite the decrease in the mitochondrial pool after UA treatment, the remaining mitochondria are robust and meet the energy requirement. A long-term UA administration in rodents induced mitochondrial biogenesis and mitophagy in the muscles of both young and old animals. The UA, at a dose of 50 mg/kg daily in aged mice which is equivalent to 4 mg/kg in humans, improved the age-related muscle decline, as well as the muscle strength suggesting its potential for treating an impaired muscle functionality. This dosing is within standard dosing regimens used for both nutrition and pharmaceutical active ingredients. Urolithins may also have different mechanisms which regulate the mitochondrial biogenesis or mitophagy, since they are known to have an estrogen receptor binding affinity. Both UA and UB regulate skeletal muscle mass also by enhancing synthesis of protein and inhibiting the ubiquitin–proteasome pathway (101). The UB can produce muscle hypertrophy and reduce muscle atrophy in mice with sciatic nerve denervation. Both urolithins, UA, and UB in different models have shown their capacity to enhance the muscle strength by different mechanisms, thus, implying its therapeutic and preventive potential in enhancing muscle strength in various pathological and age-related maladies. Given these, the ability of these molecules to act *via* estrogen receptors or how different is its action in females can be examined since estrogen deprivation, or its reduction with menopause (102) or ovarian failure, results in weakness of the skeletal muscle. Evidence points that estrogen improves the mitochondrial membrane microviscosity and the bioenergetic function in skeletal muscle (103) and in muscle proteostasis. It also increases the collagen content of tendons and ligaments. Nevertheless, it must be noted that these benefits come at the cost of a decreased connective tissue stiffness (104). It would be intriguing to know whether urolithins can take over the function of estrogen in its absence.

## Mitochondrial Regulation by Urolithins

**Figure 1:**
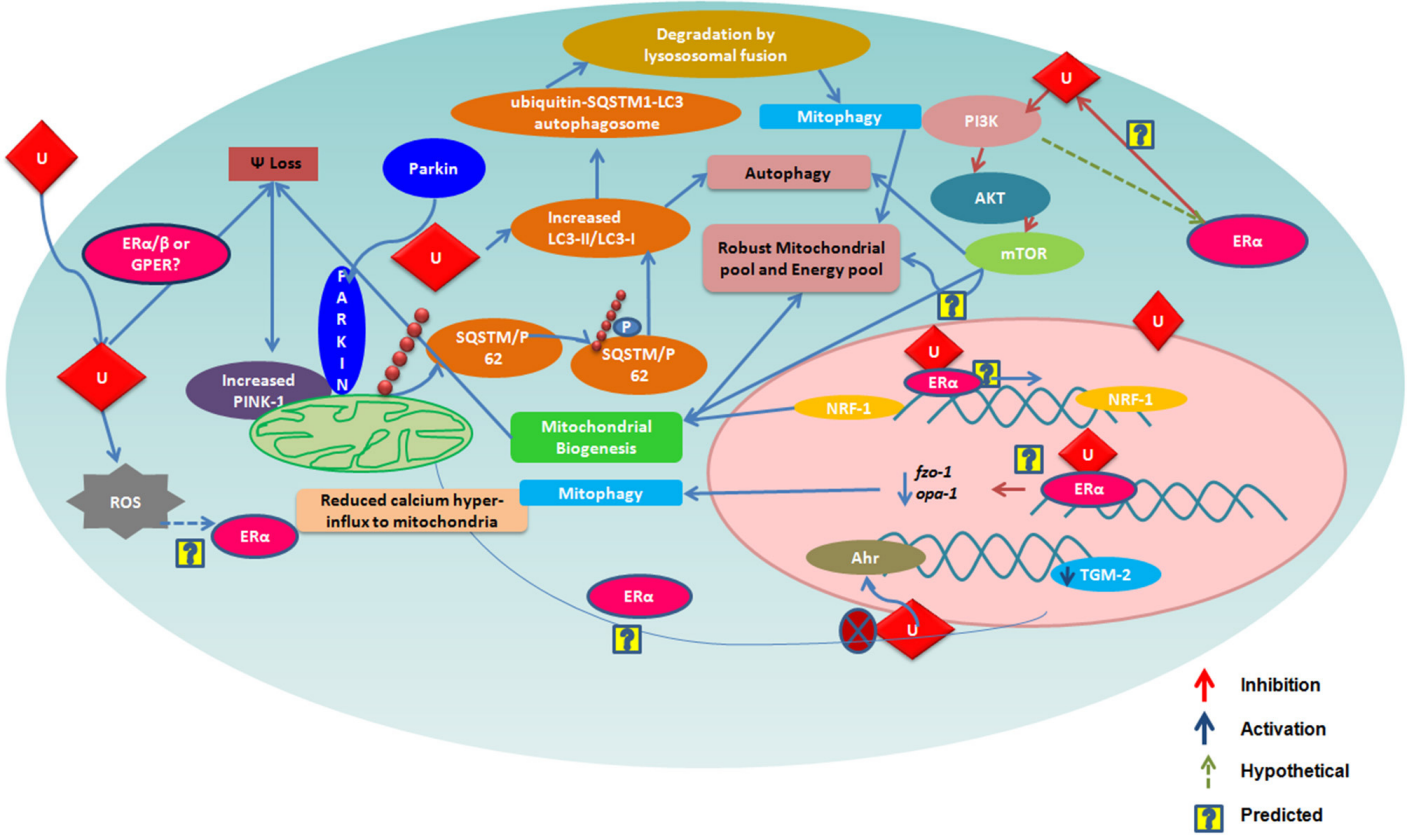
The figure depicts various mechanisms by which Urolithin A (UA) is reported to regulate mitochondrial function. Many of these are also known to be modulated *via* Estrogen Receptor α (ER α). Here, we illustarate pathways that could be mediated by urolithins *via* ER α and, hence, produce a robust mitochondrial pool maintaining a healthy pool of cells.

**Figure 2:**
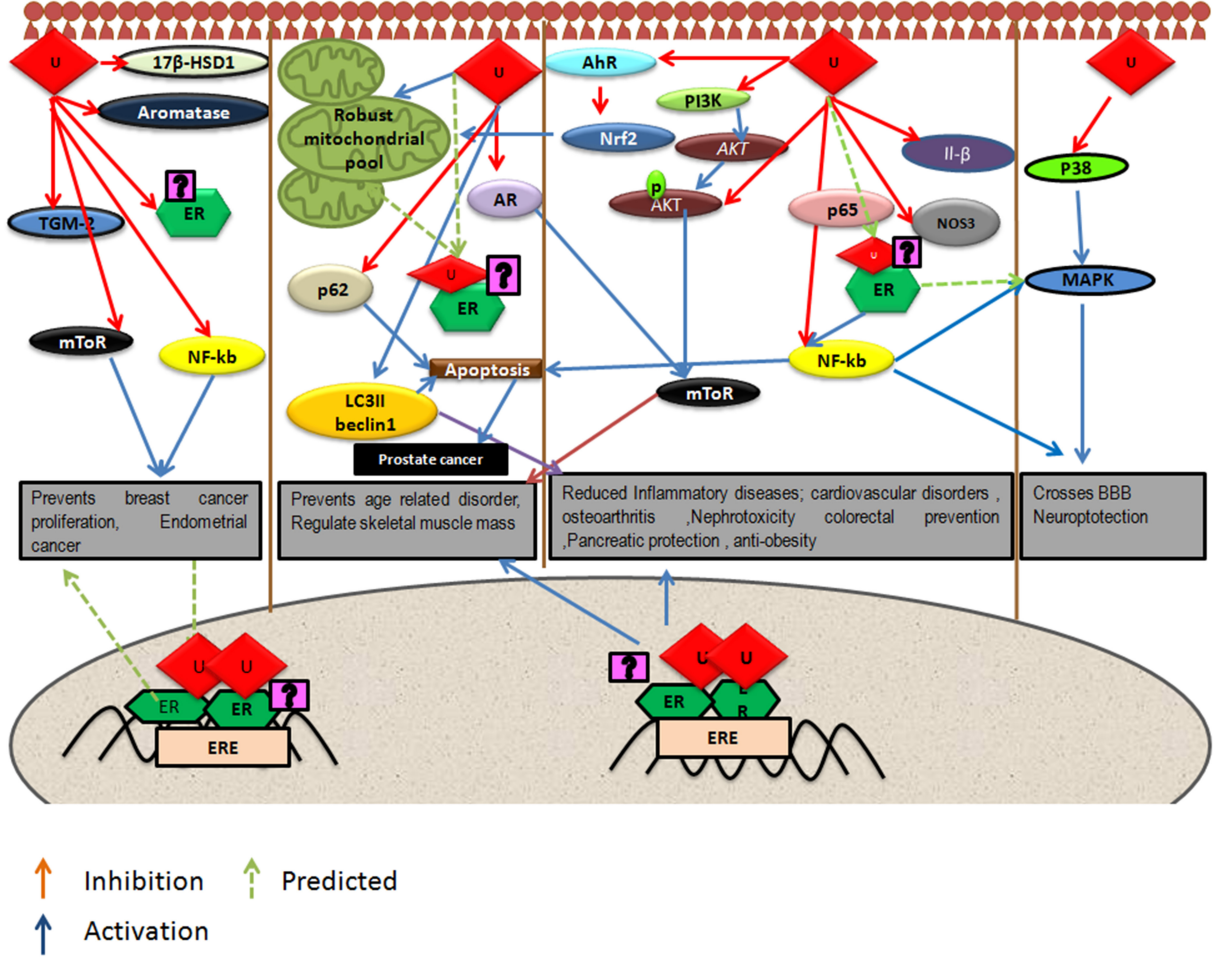
Depicts pathways by which urolithins, mainly Urolithin A and Urolithin B, can act in different tissues were U represents either UA or UB. Some of these beneficial effects might be mediated by Estrogen Receptor α (ER α) since these pathways have known to be modulated by Estrogen receptors.

Urolithins can induce mitophagy (83) and mitochondrial biogenesis in aged animals; the final consequence being the improvement of organismal phenotype and maintenance of maximum respiratory capacity emphasizing potential of urolithins having a dual role to maintain healthy mitochondria. The role of estrogen receptors in mediating this effect has not been investigated. The UA was found to induce PINK-1 and has increased the biomarkers for autophagy and mitophagy with ubiquitination of p62/SQSTM. Cells have developed sophisticated and elaborate mechanisms to adapt to stress conditions and alterations in metabolic demands, by regulating mitochondrial number and function by the generation of new mitochondria and by the removal of damaged or unwanted mitochondria for the maintenance of mitochondrial and cellular homeostasis (105). This implicates that urolithins could help in preventing many pathological conditions resulting in or caused from damaged mitochondria or failed mitochondrial metabolism. It has been reported that UA exert gut barrier functions through activation of aryl hydrocarbon receptor (AhR)-nuclear factor erythroid 2-related factor 2 (Nrf2)-dependent pathways to upregulate epithelial tight junction proteins (40). The UA inhibits transglutaminase type 2 (TGM2)-mediated mitochondrial calcium influx, which alleviates high glucose-stimulated amyloidogenesis and neuronal degeneration (106), thus, regulating mitochondrial and calcium homeostasis. Tamoxifen, a SERM, is also known to target mitochondria by ER-dependent and independent pathways. Further, the tamoxifen-resistant cells are known to display altered mitochondrial pathways with increased mitochondrial content like many other cancer drugs (107). It has been seen that drug resistance can be overcome by modulating estrogen–estrogen receptor-mitochondrial pathway. Estrogen (108) *via* ERs is involved in the life cycle of mitochondria and controls the mitochondrial biogenesis, mitochondrial quality control, and mitophagy (108, 109). Examining whether urolithins can modulate mitochondrial pathways *via* estrogen receptors can help better understand its potential. The Figure 1 consolidates the estrogen mediated mitochondrial pathways and how urolithins, through ERs could or by other mechanisms, can possibly act in a similar way. Thus, urolithins by different molecular pathways exert beneficial effects in multiple tissues, but whether these effects involve estrogen receptors remain elusive. Figure 2 shows the different pathways *via* which urolithins act and how estrogen receptor could be part of these mechanisms.

## Coronavirus Disease (COVID-19)

Clomiphene and toremifene SERMs are reported to have potent inhibitory activity in filovirus infections like EBOLA infection (110), while raloxifene hydrochloride and quinestrol inhibit flaviviruses such as Zika virus in ER-independent pathways (111). Use of SERMs in the cytokine storm and in the inflammation associated with COVID-19 is suggested as a promising pharmacological option (112). One of the main reasons that the COVID-19 is a threat to global health is due to the lack of targeted therapeutic agents. Out of the several coronavirus proteins proposed as druggable targets, 3-chymotrypsin-like protease or main protease (Mpro), a non-structural protein that breaks down the viral polyproteins to generate other non-structural proteins, the RNA-dependent RNA polymerase and helicase, is considered pivotal (113). Given this, it is interesting to note that urolithin metabolites can exert a mild anti-SARS-CoV-2 Mpro inhibition (113) at physiological relevant concentrations (2–50 μM), detectable in human colon tissues after consumption of hydrolyzable tannin-rich foods, as per clinical studies (114). Also, the pomegranate peel extract, punicalin, punicalagin, and UA have the potential to block the SARS-CoV-2 spike (S) glycoprotein receptor-binding domain (RBD)-ACE2 receptor on host cells contact, which is one of the first steps of virus infection (115). Additionally, it has been understood that SARS-Cov-2 can activate pro-inflammatory chemokines in the early stage, and this can lead to the development of either a protective immune response or an exacerbated inflammatory response (116). The latter one may lead to cytokine storm, which is clinically manifested by acute respiratory distress syndrome and systemic consequences like intravascular coagulation (116). As said in the earlier section of this review, UA exhibits anti-inflammatory activity in various tissues (39, 45, 117–119) by modulating proteins like NF-kb (44, 45), and other pro-inflammatory molecules like cytokine fractalkine (49, 120). Thus, UA manifests a natural immune-suppressant profile (121). It also protects *C. jejuni* infection in mice and protects other organs including lungs from inflammatory response (122). Furthermore, a recent study illustrated tissue deconjugation of UA-glu to UA in endotoxemia, thus, increasing free UA in systemic tissues, reaching relevant concentrations, and, hence, probably imparting a higher anti-inflammatory potential (72). In the light of these findings, urolithins with its immune modulatory and anti-inflammatory activity could be exploited to reduce such exacerbated inflammatory response as well.

## Safety Assessment

Oral administration of UA has been studied at both preclinical (27, 37, 121) and clinical level (123). Both oral and intravenous administration of urolithins showed a higher prevalence of the conjugated forms of urolithins, namely glucuronidated and sulfonated forms (27). Urolithins did not induce genotoxicity in the *in vitro* assays. The no-observed-adverse-effect-level (NOAEL) was the highest dose tested, and UA was given as 5% of the diet, or 3,451 mg/kg bw/day in males and 3,826 mg/kg bw/day in females, in the 90-day oral study. In the *in vivo* studies, the clinical parameters, blood test, or hematology did not point to any toxicity. Human randomized (123), study on the safety profile of UA in the elderly and sedentary human subjects did not show adverse effects on UA consumption in any of the oral dosing regimens. Its presence was seen in plasma and skeletal muscles. The biomarkers of mitochondrial function in the skeletal muscle and plasma metabolomics were also recorded in the study. Efforts are also being made to make urolithins more bioavailable (28, 123, 124). As mentioned earlier, the UA has also been recently recognized as GRAS for its use as an ingredient by FDA, USA. The major studies performed using urolithins has been consolidated in Table 1.

**Table 1:** The table consolidates the relevant studies related to Urolithins and their significant outcome.

**Urolithin type**	**Activity/diseasetested**	**Model**	**Concentration**	**Effects**	**Molecular target/pathway**	**Conclusion**	**Reference and year**
UA and UB	Estrogenic/Anti-estrogenic	MCF-7	0.1–40 μm	Proliferative, but prevents E2 induced proliferation. Binds to ERα and ERβ with different affinity. UA has higher affinity	Estrogen receptors	UA and UB have estrogenic and anti-estrogenic activity	Larrosa et al., 2006 (19)
UA	Colon inflammation	Male Fischer rats induced with acute colitis by dextran sodium sulfate	UA:15 mg kg/ day	Pomegranate extract and UA decreased inflammation markers and favorably modulated the gut microbiota. UA preserved colonic architecture	UA decreased inflammation markers like iNOS, cycloxygenase-2, PTGES. and PGE2 in colonic mucosa	UA probably the most active anti-inflammatory compound derived from pomegranate ingestion in healthy subjects, while in colonic inflammation group the effects may be by non-metabolized ellagitannin-related fraction	Larrosa et al., 2010 (37)
UA, UB, mUA, mUB	Alzheimer's disease	*In silico, C. elegans*	10–100 μM 10 μg/ml for 40 h	Urolithins passes BBB criteria, urolithin reduces Aβ fibrillation mUB protected C.elegans from Aβ induced neurotoxicity and paralysis	NA	Urolithins can reduce Aβ fibrillation	Yuan et al., 2015 (94)
UA	Prostate cancer	LNCaP cell line	40 μM	UA increase cells in G1-phase, induction of apoptosis	UA upregulates CDKN1A	A potential chemopreventive agent for prostate cancer	Sánchez-González et al., 2016 (79)
UA and UB	Endometrial cancer	ECC1, Ishikawa cell	0.1–50 μm	Antiproliferative, G2/M arrest, ERα modulation	Cell cycle proteins, suppresses ERα, enhances ERβ, and PGR, Ps2, GREB1, down GRIP1	Antiproliferative in in endometrial cancer	Zhang et al., 2016 (20)
UA, UB, UC, and UD	Lifespan extension	Bacteria: *E. coli*, C2C12 myoblast, K intestinal cell *C. elegans*, Sprague-Dawley rats C57BL/6J	25–50 μM, 8 h 10–50 μM,4–24 h 50 μM till death, 25 mg/kg/d−7days, 50 mg/kg/d−34 weeks	Mitophagy induction Improved pharyngeal pumping rate and mobility, better maintenance of muscle fiber organization, mitophagy induction, decreased mitochondrial content while maintaining maximum respiratory capacity, long term exposure induced mitochondrial biogenesis	UA lowered *fzo-1* and *opa-1*: Important in mitochondrial fusion machinery UA acted via genes *bec-1, sqst-1* and *vps-34*, and the mitophagy genes *pink-1, dct-1* and *skn-1* (Nrf2) homolog	UA improves mitochondrial and muscle function	Ryu et al., 2016 (83)
UA, UB, UC	Pheochromocytoma	PC12 cells	10–300 μg/ml	UC treatment increased lactate dehydrogenase release and membrane lipid peroxidation, and induced cell apoptosis, cell cycle arrest at S phase, and Reactive oxygen species (ROS)	Apoptosis Pathway: Bcl-2/Bax caspase 9 and caspase 3	UC, showed potent cytotoxicity in PC12 cells compared to EA	Yin et al., 2017 (125)
UA and UB	Diabetic cardiomyopathy	Wistar rats induced with type-II diabates	2.5 mg/kg /day: IP 3 weeks	Prevented early response of cardiac cells to hyperglyceamia, improved myocardial microenvironment, and maximal rate of ventricular pressure rise, recovery of cardiomyocyte contractility, and calcium dynamics	SERCA2/PLB Ratio increase and Reduced CX3CL1 when compared to diabetic group	Prevents the initial inflammatory response of myocardial tissue to hyperglycemia	Savi et al., 2017 (49)
UA and UB	Toxicity study	Human peripheral lymphocytes Wistar rats	0.0006–2.29 mg/ml 1,000 mg/kgw oral, 2.5 mg/kg bw i.v	No changes or frameshifts No gene mutations by base pair 28- and 90-day study: Non-genotoxic, no change in clinical chemistry, hematology, or urine analysis. No toxicity observed at any target organ	NA	The NOAEL was the highest dose tested,5% UA by weight in the diet, or 3,451 mg/kg bw/day in males and 3,826 mg/kg bw/day in females	Heilman et al., 2017 (27)
UA UA, UB	Skeletal muscle mass	C2C12 myotubes Twelve-week-old male or female C57/Bl6 J mice	15 μM 10 μg/day of urolithin B during 28 days	UB not UA enhances differentiation of C2C12 myotubes UB induces muscle hypertrophy, reduces muscle atrophy	Represses ubiquitin proteasome pathway. crosstalk between the AR and the mTORC1 pathway, possibly via AMPK	UB has potential for the treatment of muscle mass loss	Rodriguez et al., 2017 (101)
UA and EA	Cisplatin-induced nephrotoxicity	Male Sprague Dawley rats	50 mg/kg body weight-5 days	UA reduced creatinine and tubular apoptotic cells in Cisplatin-induced kidney damage Reduced macrophage infiltration	Reduced NF-kb and NOS3, Iba1 induced by cisplatin in kidney	UA mitigates cisplatin-induced nephrotoxicity in rats	Guada et al.,2017 (117)
UA, UB, UC	Prostate cancer	LNCap cells	10–40 μM	Urolithins inhibited proliferation of LNCaP prostate cancer cells. The mixtures of bicalutamide with UA and UB had additive anti-proliferative effect. Combinations of bicalutamide with UA and UB had attenuated pro-apoptotic activity	NA	The differences in activity of urolithins in prostate cancer imply health benefits and interactions will depend on the type of produced ellagitannins metabolite	Stanisławska et al.,2018 (80)
UA	Anti-inflammatory potential in macrophages	J774.1 murine macrophage HEK,293 cell lines	1–50 μM	UA strong inhibitor of M1 (LPS) macrophage polarization, UA elevates autophagic flux in macrophages	Inhibit p65 nuclear transclocation Reduced pro-inflammatory proteins and NO production Impaired Akt/ mTOR signaling	Increased activity of the autophagic cellular recycling machinery aids the anti-inflammatory bioactivity of UA	Boakye et al., 2018 (118)
UA	Colorectal cancer	SW620	1–30 μM	UA decreased cell proliferation, and cell migration, Induced autophagy, and apoptosis. Suppressed cell cycle progression	Induced LC3	UA induces autophagy and inhibit CRC cell growth and metastasis	Zhao et al., 2018 (126)
UA, UB, Iso-UA, and UA conjugates	Breast cancer	MCF-7 MDA-MB-231	1–50 μM	Alycones exerted antiproliferative and estrogenic/antiestrogenic activities but both their glucuronide and sulfate conjugates lacked these activities	NA	Antiproliferative and estrogen receptor modulatory activity in breast cancer cells	Ávila-Gálvez et al., 2018 (58)
UA	Effect on immune cells	Murine CD4+ T cells	5–50 μM	UA regulaes of Ca^2+^ entry into CD^4+^ T cells leading to suppression of CD^4+^ T cell activation	Upregulates the expression of miR-10a-5p which in turn decreases store-operated Ca^2+^ entry (SOCE), by downregulating Orai1 and STIM1/2 expression	UA could be used a natural immune suppressant during various inflammatory disorders including inflammatory bowel disease	Zhang et al., 2019 (121)
UA	Alzhimers disease (AD)	PPswe/PS1ΔE9 (APP/PS1) mouse model of AD	300 mg/kg	UA ameliorated cognitive impairment, prevented neuronal apoptosis, and enhanced neurogenesis, attenuated Aβ deposition, and peri-plaque microgliosis and astrocytosis in the cortex and hippocampus	UA enhanced cerebral AMPK activation, decreased P65-NF-κB activation and P38MAPK, and suppressed Bace1 and APP degradation	UA imparted cognitive protection by protecting neurons from death and triggering neurogenesis via anti-inflammatory signaling	Gong, 2019 (96)
UA	Tissue deconjugation of UA	LPS administered male Sprague-Dawley rats	26 mg / kg b.w	Tissue deconjugation of UA-glur to UA after lipopolysaccharide (LPS)-induced inflammation	NA	Tissue deconjugation of Uro-A glur to UA after lipopolysaccharide (LPS)-induced inflammation, explaining systemic *in vivo* activity of free Uro-A in microenvironments subjected to inflammatory stimuli	Ávila-Gálvez et al., 2019 (72)
UA	Mitochondrial and cellular health	Healthy, sedentary elderly individuals	1,000–2,000 mg of UA delivered orally	UA has a favorable safety profile UA bioavailable in plasma modulated plasma acylcarnitines and skeletal muscle	Mitochondrial gene modulation	UA induces a molecular signature of improved mitochondrial and cellular health	Andreux et al., 2019 (123)
UA and synthetic analog UAS03	Beneficial activities at gut epithelium	HT29 bone marrow derived macrophages Male mice (C57BL/6J; 6–8 weeks old)	Oral doses 20 mg/kg at 6–24 h	Anti-inflammatory activities and enhanced gut barrier function	Activation of aryl hydrocarbon receptor (AhR) (Nrf2)-dependent pathways to upregulate epithelial tight junction proteins	Attenuated colitis in pre-clinical models by remedying barrier dysfunction in addition to anti-inflammatory activities	Singh et al., 2019 (40)
UA	Increase availability by nanoparticle encapsulation	Male Sprague/Dawley rats	Oral gavage a single dose of 50 mg plain UA, 25 mg P2Ns UA, or 10 mg or 25 mg P2Ns-GA UA	Nanoparticle encapsulated UA led to a seven-fold enhancement in oral bioavailability. It attenuated the histopathological hallmarks of cisplatin-induced AKI and reduced mortality by 63%	Nanoparticle UA therapy downregulated Nrf2 and P53-inducible genes and involved anti-apoptotic signaling	Nanoparticles greatly increase the oral bioavailability of UA leading to improved survival rates in AKI mice, in part by reducing renal oxidative and apoptotic stress	Zou et al., 2019 (124)
UA	Type 2 diabetes	Type 2 diabetes model was induced by HFD; and streptozotocin (85 mg/kg)	UA (50 mg/kg/d) alone or UroA-chloroquine combination for 8 weeks	UA improved symptoms of diabetic mice, pancreatic function indexes. UA decreased mitochondrial swelling and myelin-like cytoplasmic inclusions	Upregulated light chain 3-II (LC3II) and beclin1, downregulated sequestosome 1 (p62), and decreased apoptotic protein cleaved caspase3 partly by (p-Akt)-p-mTOR pathway	UA protects pancreas against diabetes	Tuohetaerbaike et al., 2020 (120)
UA	Osteoarthritis	Primary chondrocytes *Ex vivo* organ culture of articular cartilage	1–15 μM 1–7 days	No UA cytotoxicity UA protected IL-1β induced cartilage damage. UA protective in *ex vivo* organ culture of articular cartilage	UA protected chondrocytes against IL-1β-induced injury by activating the mitogen-activated kinase (MAPK)/nuclear factor-κB (NF-κB) signaling pathways	UA attenuated IL-1β-induced cell injury in chondrocytes via its anti-inflammatory action	Ding et al., 2020 (45)
UA	Obesity	Six-week-old male C57BL/6 mice 4-week-old male leptin-deficient *ob*/*ob* mice	30 mg kg	UA increases energy expenditure by enhancing thermogenesis in brown adipose tissue and inducing browning of white adipose tissue	UA enhances adipose tissue production of triiodothyronine (T3), which activates thermogenic genes PGC1a and UCP-1	UA suggested as potent anti-obesity agent	Xia et al., 2020 (67)
UA	Alzheimer's disease	SH-SY5Y cells Streptozotocin (STZ)-induced diabetic mouse model	UA:100 nM UA:2.5 mg/kg/day: 8 weeks	UA prevented Aβ-induced mitochondrial calcium influx, mtROS accumulation, Tau phosphorylation, and cell death in neuronal cells	UA significantly reduced high glucose-induced TGM2 expression and disrupted AIP–AhR complex.	UA may prevent diabetes mellitus associated AD pathogenesis by reducing TGM2-dependent Mitochondria-associated membranes (MAM) formation and maintaining mitochondrial calcium and ROS homeostasis	Lee et al., 2020 (106)
UA	Campylobacteriosis	Abiotic IL-10^−/−^ mice infected with *C. jejuni*	0.114 mg /kg/B.W/day	UA lowered pathogen loads in ileum, but not colon. Improved clinical outcome and less inflammatory sequelae of infection. Reduced intestinal and systemic pro-inflammatory immune responses	Lowered IFN-γ, TNF-α	Oral UA administration is a promising treatment option for acute *C. jejuni* infection	Mousavi et al., 2021 (122)
UA UB urolithin glucuronides	Anti-inflammatory activity	THP-1-derived macrophages, RAW 264.7 macrophages	40 μM	UA was the most active metabolite in terms of LPS-induced inflammatory response inhibition	Attenuate NFκB p65 nuclear translocation, and stimulate ERK1/2 phosphorylation	UA the most potent in inflammatory response	Bobowska et al., 2021 (16)
UA mUA UB	SARS-CoV-2, main protease (Mpro) inhibitors	Assay kit consisting of recombinant Mpro	2–50 μM	Urolithins inhibited severe acute respiratory syndrome corona virus (SARS-CoV-2) SARS-CoV-2 Mpro (by 6.6–100.0%) and bound directly to the Mpro protein	Inhibition of Mpro	Inhibitory effects of tannins and their metabolites on SARS-CoV-2 Mpro	Li et al., 2021 (113)

*The UA denotes Urolithin A, UB Urolithin B and UC Urolithin C. The mUA is methylated urolithin A, while the mUB is urolithin B*.

## Conclusion

To summarize what has been discussed, so far, we propose that urolithins could be beneficial in general wellness and health. Its relevance seems more pronounced in the hormone-dependent tissues, which connote its potential in hormone or endocrine-related pathogenesis. A plethora of evidence pointedly illustrate the health benefits of urolithins in cardiovascular health, muscle strengthening, bone health, breast and endometrial cancer, aging, brain related diseases, and pathologies stemming from an inflammatory response or its consequence like in the case of COVID-19 infection. Many of these may involve the hormone receptor estrogen receptors along with the other pathways. The mechanism of action of urolithins, mediated *via* estrogen receptors, is very sparsely studied. Competitive binding studies and transactivation assays point to its ability to act as an estrogen agonist. However, it is known that estrogen receptors exhibit a complex and dynamic activity depending on the different conformation it attains according to the ligand structure and binding. It depends on the tissue it acts since the co-factors available and recruited by estrogen receptors vary between the cell types. Albeit estrogen receptor agonists, antagonists and SERMS can activate or repress unique genes, they can also trigger or repress similar subset of genes. Ergo, urolithins need to be examined for its responses in different hormone responsive tissues; its potential as estrogenic and endocrine disruptors, and whether the known health benefits involve an ER-mediated action. Urolithins, or its source, which include ellagitannin-rich food like pomegranate and the bacteria responsible for its production, could also serve as supplement as probiotics. Also, studies can be undertaken to illustrate the potential of urolithins at a clinical level on how these molecules would act in combination with an already known synthetic SERM. Nonetheless, due to pleotropic nature of estrogen receptors, it is important to consider the potential long-term merits and the adverse effects of urolithins in the estrogen receptor-dependent tissues. Taking together the recent research on urolithins, we propose this could serve as an endocrine modulator and that further investigations in this direction need attention.

## Author Contributions

RV and SS conceived the idea of the article. RV and VR screened and retrieved the data and prepared diagrams. RV prepared the manuscript draft. RV, AS, TS, RA, PM, and LL tabulated various studies. SS reviewed and corrected the manuscript. All authors read and approved the final manuscript.

## Funding

RV and VR acknowledge Indian Council of Medical Research (ICMR) for Senior Research Fellowship (SRF). The study was supported by the Science and Engineering Research Board, Department of Science and Technology (SERB-DST) (No. EMR/2016/000557).

## Conflict of Interest

The authors declare that the research was conducted in the absence of any commercial or financial relationships that could be construed as a potential conflict of interest.

## Publisher's Note

All claims expressed in this article are solely those of the authors and do not necessarily represent those of their affiliated organizations, or those of the publisher, the editors and the reviewers. Any product that may be evaluated in this article, or claim that may be made by its manufacturer, is not guaranteed or endorsed by the publisher.
